# Dose‐Dependent Effects of Lithium on Biological Pathways, Ribosomes, Mitochondria, and Proteasomes in Hippocampal Neurons: Insights from Gene and Protein Expression

**DOI:** 10.1002/alz70855_103806

**Published:** 2025-12-23

**Authors:** Orestes Vicente Forlenza, Vanessa J R De Paula, LIm‐27 Neuroscience

**Affiliations:** ^1^ University of Sao Paulo, Sao Paulo, SP, Brazil; ^2^ Departamento e Instituto de Psiquiatria, SP, 05403‐903, Brazil

## Abstract

**Background:**

Lithium salts have a well‐established role in the treatment of psychiatric disorders, such as major affective disorders, and more recently Alzheimer disease. Experimental and clinical studies have provided evidence that lithium may exert neuroprotective effects at therapeutic and subtherapeutic doses. In animal and cell culture models, lithium has been shown to increase neuronal viability through a combination of mechanisms that includes the inhibition of apoptosis, regulation of autophagy, increased mitochondrial function, and synthesis of neurotrophic factors. The aim of this study is to compare biological processes and pathways enriched in embryonic hippocampal neurons treated with different doses of lithium (0.02mM, 0.2mM and 2mM), integrating differentially expressed genes and proteins.

**Method:**

Animal experiments were approved by the animal care ethics committee of the University of Sao Paulo, Brazil, and all national guidelines were taken into consideration. Hippocampal neurons were cultured from day 18 Wistar rat embryos and treated with lithium (0.02mM, 0.2mM, and 2mM) from day 7 for 4 days. Gene expression was analyzed via microarray, identifying differentially expressed genes (13 at 0.02mM, 23 at 0.2mM, and 53 at 2mM), while proteomics detected 46 proteins at 0.02mM, 16 at 0.2mM, and 15 at 2mM. Data integration using STRING protein interaction networks revealed key biological processes affected by lithium, offering insights into its molecular mechanisms and potential therapeutic effects.

**Result:**

The results showed that growing gene networks using differentially expressed genes and proteins as seeds alterations in mitochondria, ribosome and proteasome were observed for all lithium treatment doses. Additionally, more specific biological processes such as mitochondrial respiratory chain, energy production (glucose and ATP) and apoptosis were enriched in each dose treatment. Interestingly, it seems that low doses are more related to cytoplasmic or specific organelle functions, while high doses are related to nuclear activity.

**Conclusion:**

There is no dissociation dose effect of the treatment with lithium, the dose of 0.02Mm modified genes of degradation pathway proteins. The dose of 0.2Mm altered pathways of energy metabolism and the dose of 2Mm change related to nuclear and nuclear membrane. Thus, we can understand the specific function of each dose of the treatment.

